# Intravenous Vitamin C Supplementation in Allogeneic Hematopoietic Cell Transplant Recipients: Salutary Impact on Clinical Outcomes

**DOI:** 10.21203/rs.3.rs-3538792/v1

**Published:** 2023-11-10

**Authors:** Amir Toor, Gary Simmons, Roy Sabo, May Aziz, Erika Martin, Robyn Bernard, Manjari Sriparna, Cody McIntire, Elizabeth Kreiger, Donald Brophy, Ramesh Natarajan, Alpha Fowler, Catherine Roberts

**Affiliations:** Virginia Commonwealth University Massey Cancer Center; Virginia Commonwealth University Massey Cancer Center; VCU; VCU; Virginia Commonwealth University; Virginia Commonwealth University; Virginia Commonwealth University; Virginia Commonwealth University; Virginia Commonwealth University; Virginia Commonwealth University; Virginia Commonwealth University

**Keywords:** Parenteral ascorbic acid, allogeneic stem cell transplantation, graft vs. host disease, myeloid malignancy, non-relapse mortality

## Abstract

Intravenous (IV) vitamin C improves organ function and reduces inflammation in sepsis, an inflammatory state like the post-hematopoietic stem cell transplant (HCT) milieu. The safety and efficacy of parenteral vitamin C after allogeneic HCT were evaluated in a phase I/II trial. Clinical outcomes were compared with a propensity score - matched historical control.

**Methods:**

Patients with advanced hematologic malignancies received IV vitamin C, 50mg/kg/d, divided into 3 doses given on days 1–14 after HCT, followed by 500 mg bid oral from day 15 until 6 months post-SCT.

**Results:**

55 patients received IV vitamin C. All patients were deficient in vitamin C at day 0. Vitamin C recipients had lower non-relapse mortality (NRM) (p = 0.07) and improved survival compared to historical controls (p=0.06), with no attributable grade 3 and 4 toxicities. Vitamin C recipients had similar relapse rate and acute graft versus host disease (GVHD) (p=0.35), but lower severe chronic GVHD (p=0.35). Patients with myeloid malignancies had improved survival (p=0.02) and NRM (p=0.009), as well as chronic GVHD, with similar relapse rates compared to controls.

**Conclusions:**

In patients undergoing allogeneic HCT the administration of IV vitamin C is safe and reduces non-relapse mortality and chronic GVHD improving overall survival.

## INTRODUCTION

Hematopoietic stem cell transplant (HSCT) remains a potentially curative therapy provided by the high dose intensity of the preparative regimens used and the immune mediated graft vs leukemia effect^1^. High-dose preparative regimens such as myeloablative condition with ionizing radiation and alkylating agents such as busulfan or melphalan, lead to considerable epithelial and endothelial toxicity. These regimens induce variable degree of enteral mucositis, loss of functioning epithelial cells and disrupt tight junctions increasing the translocation of bacterial products and inflammatory cytokines, which is considered an essential factor in the pathogenesis of acute graft vs host disease GVHD.^2^ Further, tissue injury leads to endogenous tissue antigen presentation provoking GVHD.^3^ This contributes to 1-year transplant related mortality (TRM) in myeloablative allogeneic HSCT, which has been reported between 15–31 %.^4 5^

It is critical that therapy to mitigate endothelial and tissue injury in HSCT recipients be developed to aid in balancing the competing hazards of tissue injury and GVHD leading to TRM. One agent that may serve this purpose is ascorbic acid, vitamin C. Ascorbic acid is an effective anti-inflammatory agent with demonstrated ability to inhibit NF-kB-driven inflammatory cytokine (IL-6, IL-8, and TNF-α) expression, and by its ability to attenuate endothelial permeability.^6 7^ Further, hypovitaminosis C is well described in critically ill patients, who have several parallels with HSCT patients, such as, elevation of biomarkers including C-reactive protein (CRP), as well as having elevated levels of inflammatory cytokines. In sepsis patients treated with parenteral vitamin C, organ function improves and inflammatory markers decline.^8^

In previous reports, vitamin C deficiency was prominent throughout the acute phase of HSCT and this was significantly associated with high inflammatory markers, CRP, and ferritin.^9^ Vitamin C administered in enteral nutrition may not be adequate due to nominal levels.^10^ Previous work in a prospectively studied cohort of patients undergoing myeloablative conditioning allogeneic HSCT, found patients to be uniformly vitamin C deficient and these remained deficient up to 60 days post-transplant. Patients with severe mucositis had lower vitamin C levels at day 14 after HSCT.^11^

Recognizing that vitamin C has anti-inflammatory properties and patients undergoing allogeneic HSCT are deficient in this critical nutrient likely due to increased consumption and reduced dietary intake and absorption, a phase I-II study was developed to administer parenteral vitamin C following transplantation. This was done to study the hypothesis that mitigating the pro-inflammatory effects of HSCT with early administration of vitamin C in patients deficient in this micronutrient will attenuate endothelial and organ injury from high-dose conditioning and ameliorate GVHD and transplant-related mortality. The results of this trial are now reported, comparing patients who received parenteral vitamin C after myeloablative conditioning for allogeneic HSCT to propensity-matched historical controls.

## METHODS

### Patients

This was a prospective phase I/II clinical trial designed to study the impact of parenteral vitamin C administration in recipients of allogeneic SCT (FDA_IND 138924). This trial was approved by the Massey Cancer Center Protocol Review Committee and Human Subjects Institutional Review Board (IRB) at Virginia Commonwealth University (VCU). All patients signed IRB-approved informed consent forms and were treated at VCU (NCT03613727). The study population included adult patients 18–78 years old with acute myelogenous leukemia (AML), acute lymphoblastic leukemia (ALL), chronic myelogenous leukemia (CML), and myelodysplastic syndrome (MDS) who underwent their first allogeneic stem cell transplant from matched sibling donors and unrelated donors who were matched at either 7/8 or 8/8 HLA-A, -B, -C, - DRB1 loci using high-resolution DNA-based typing. Patients undergoing non-myeloablative conditioning were not included.

### Vitamin C administration

An initial cohort of patients (N=14) were enrolled if they had low vitamin C levels (<0.5mg/dl). Vitamin C levels were checked at baseline and on day −2 in these patients. Patients were treated with parenteral vitamin C 50 mg/kg/d (Ascorbic Acid; McGuff Pharmaceuticals, Santa Ana, CA) divided in three doses from day +1 through day +14 and transitioned to oral vitamin C 500 mg twice daily from day +15 through day +180 (**Supplementary Figure 1**). Vitamin C was given in 50 mL of 5% dextrose and water over 30 minutes every 8 hours in UV-light-protected bags and tubing. Subsequently consecutive patients meeting above criteria were enrolled in the phase II part of the trial till study completion (N=55).

### Cytokine analysis

Vitamin C and CRP were checked before conditioning (baseline) and on the following days: day 0, 14, 30, 100 in all the patients. In the first 14 patients enrolled pro-inflammatory cytokines IL-1b, IL-2, IL-6, IL-12, TNF-α, IFN-γ, as well as soluble thrombomodulin were measured at baseline and on days 0, 14 and 30; IL4, IL-5, IL-10, IL-13, IL-17A/CTLA8, IL-21, IL-22, IL-23, were also measured at these times. Cytokines were assayed in patient plasma with multiplex technology using the xMAP Luminex 200 (Luminex Corp, Austin TX). These data were displayed in heat maps where, to normalize the cytokine measurements, the cytokine levels at each time point in each patient (days 0, 14, 30, 100) were divided by the corresponding baseline measurements for that patient. Further grouping of the resulting data was completed via conditional formatting in MS Excel into low (green), medium (yellow) and high (red) valued cytokine levels to give a visual representation of change over time.

### Statistical Analysis

The primary endpoint was non-relapse mortality at one year and secondary endpoints included engraftment, acute and chronic GVHD, relapse, and overall survival. Simon’s two-stage design was utilized for this study. In the first stage, a threshold of ≥35% NRM was set to result in trial termination for futility. The total sample size calculated for Simon’s two-stage design was 55 patients for a reduction in NRM from 35% to 20%, a 42% relative reduction compared to a propensity-matched historical control cohort. Propensity score matching was performed to identify similarly treated patients transplanted from January 2015 to December 2018. A logistic regression was fit with the group as the binary outcome (Intervention vs. Historical Control) against three matching variables, including diagnosis (ALL, AML, and CML+MDS), conditioning regimen (busulfan + cyclophosphamide, Fludarabine + Melphalan, and total body irradiation+Cyclophosphamide), and disease risk (High and Intermediate+Low). Disease risk was defined by the CIBMTR risk index. The resulting probabilities of belonging to the Intervention group were used as propensity scores. Nearest neighbor matching was used to identify the matching set of historical control subjects in a 1:1 ratio with the trial enrollees. CMV reactivation was defined as > 200 IU/mL; EBV > 500 and requiring Rituximab

A Kaplan-Meier step function was performed for mortality assessment, while cumulative incidence curves were constructed for relapse (accounting for competing risk of mortality), acute graft versus host disease (GVHD), chronic GVHD (accounting for competing risks of relapse and mortality), non-relapse mortality (NRM), and non-GVHD-relapse mortality (NGRM). Cox-proportional hazard models were used to estimate adjusted hazard ratios between the time-to-event health outcomes and group, adjusted for patient age, donor type, stem cell source, disease type, conditioning regimen, and disease risk. A subset analysis was performed analyzing outcomes in patients with AML, MDS and CML. The R (4.1.2) and RStudio (version 2022.02.0) statistical software platforms were used for all data management and analysis.

## RESULTS

### Vitamin C following allogeneic SCT

Patients were prospectively enrolled in the study between October 2018 – October 2021 and propensity scores matched with historical controls transplanted between 2015–2018. Follow up was updated as of February 2023. Patient characteristics are described in Table 1; 55 patients received IV vitamin C, including 10/10 HLA-MRD and MUD (n=48) and 9/10 HLA MUD recipients (n=7; 4 HLA-DQ, 2 HLA-B and 1 HLA-A mismatch). The number of fludarabine and melphalan recipients was higher in the vitamin C group as compared to historical controls. A safety lead-in cohort of 14 patients was initially enrolled. All patients enrolled were deficient in vitamin C at day 0, median level 0.3 mg/dL (range: 0.1–0.5); the median neutrophil recovery was by 12 days (range 9–15 days); similarly, platelets engrafted by day 12 (8–21 days) as well, with 100% donor myeloid engraftment at day 30. Safety endpoints of myeloid engraftment, grade 3 acute GVHD and NRM were evaluated in these patients, without triggering any of these endpoints allowing enrollment to continue.

### Engraftment in vitamin C recipients

In the patients treated with Vitamin C (N=55), the median time to neutrophil and platelet engraftment were 11 and 12 days respectively (neutrophils: 9–15 days platelet 8–21 days). T cell chimerism at day 30, 60, and 90 following HSCT was > 95% in the Vitamin C group. Concomitantly measured median CD3+ cell count on days 30, 60, and 90 were 236 (2–2024), 485 (5–5580) and 390 (112–2565)/mL respectively and were not different compared historical controls. Median CD3+/4+ cell count on day 30 was 80/mL (range 30–416), CD3+/8+ was 198/mL (25–1924), CD19+ cell count was 8/mL (range 5–84) and CD3−/56+ cell count was 260/mL (range 25–656), indicating early T and NK cell recovery in these patients.

### Clinical outcomes in vitamin C recipients

**Figure 1:**
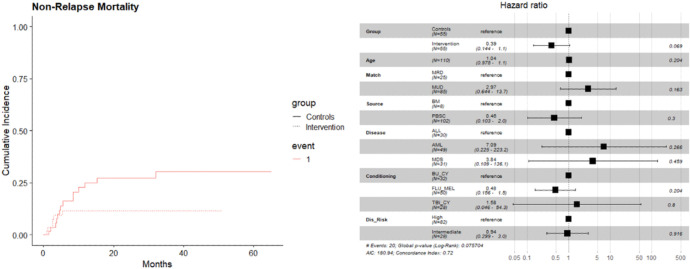
(A) CI curves depicting lower NRM in the VC treated patient. (B) In a multivariate model this was not significant (HR = 0.4, 95% CI: 0.1, 1.0, p-value = 0.069)

**Figure 2:**
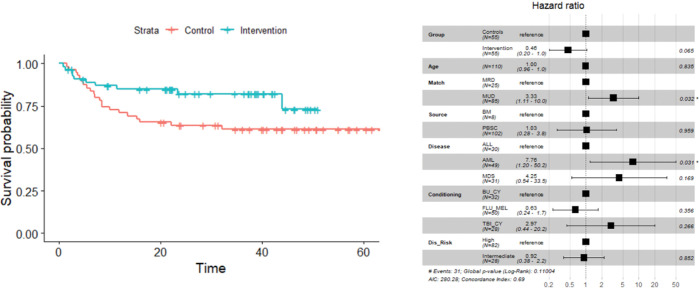
(A) KM curves depicting improved survival in the VC treated patients (p= 0.057). (B) In a multivariate model, only donor type (MUD vs. MRD: HR = 3.3, 95% CI: 1.1, 10.0, p = 0.032) and diagnosis (AML vs. ALL: HR = 7.75, 95% CI: 1.2, 50.2, p = 0.031) were significant.

Non-relapse mortality was lower in the vitamin C treated group (11%) compared to the historical control (25%), but was not statistically significant in the multivariate model (HR = 0.4, 95% CI: 0.1, 1.0, p-value = 0.069) (Figure 1). Overall survival was improved in study participants ((HR = 0.5, 95% CI: 0.2, 1.0, p-value = 0.065) (Figure 2); AML patients experienced significantly worse outcomes in multivariate analysis. Overall relapse rate was similar between the two cohorts.

The vitamin C cohort had seven HLA mismatched unrelated donors and despite this there was no increase observed in the risk of acute GVHD in the control vs. study cohorts (grade II-IV, (33%) vs. (33%), p-value = 0.81 or III-IV, (24%) vs. (17%), p-value = 0.35). Organ distribution of acute GVHD grade 3–4 in vitamin C recipients was; skin only (2), GI (3), skin and liver (1), skin and GI (2) and steroid refractory GI (2). Moderate to severe chronic GVHD rates when accounting for competing risks of relapse and mortality were lower in the Vitamin C group (11%) compared to the historical controls (24%), (adj HR = 0.60, 95% CI: 0.20, 1.76, p-value = 0.352) (**Supplementary Figure 2**).

In the vitamin C vs. historical control cohorts (7 vs. 14%, P=0.03) received rabbit anti-thymocyte globulin (ATG) as a part of GVHD prophylaxis regimen. Of note the schedule on which ATG was administered was predominantly day −3 to day −1 in the historical control and day −9 thru −7 in the Vitamin C recipients, with similar rates of acute and chronic GVHD observed despite the earlier administration of ATG, and a less profound *in vivo* T cell depleting effect.

### Clinical outcomes in myeloid malignancy

**Figure 3:**
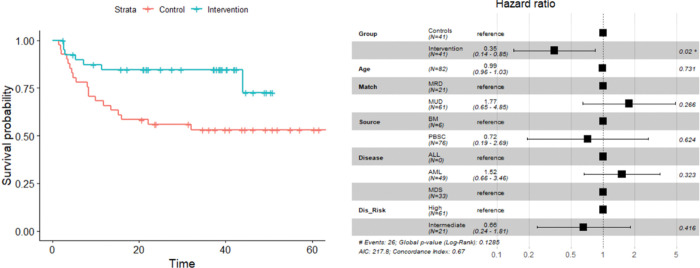
(A) KM curves depicting improved survival in the VC treated patients with myeloid malignancies (p= 0.0167). (B) In a multivariate model this relationship remained significant (HR = 0.32, 95% CI: 0.12, 0.84, p-value = 0.02) amongst myeloid malignancy patient

**Figure 4:**
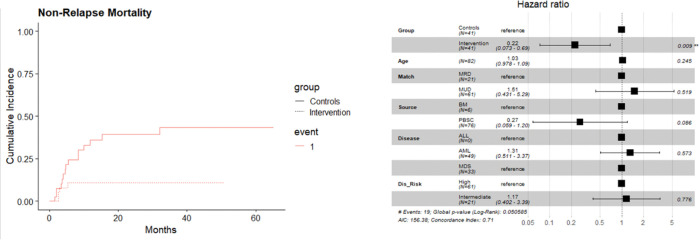
(A) CI curves depicting lower NRM in the VC treated patients with myeloid malignancies. (B) In a multivariate model this was significant (HR = 0.22, 95% CI: 0.07, 0.69, p-value = 0.009)

Because of the frequency of DNA hypermethylation in myeloid malignancies, the clinical outcomes in patients with AML, MDS and CML were examined comparing trial patients with their propensity-matched historical controls (Table 2). Overall survival was superior in these patients amongst the vitamin C recipients (p = 0.0167) (HR = 0.32, 95% CI: 0.12, 0.84, p = 0.02) (Figure 3). This was attributable to a lower risk of NRM in the vitamin C treated patients (10%) than historical controls (37%) (HR = 0.22, 95% CI: 0.07, 0.69, p-value = 0.009) (Figure 4). Relapse and acute GVHD were not different in this subgroup, however the rate of chronic GVHD tended to be lower in Vitamin C recipients (12 vs. 24%; adj HR = 0.56, 95% CI: 0.17, 1.82, p-value = 0.383) between the two groups.

### Infections and toxicities in the Vitamin C recipients

CMV reactivation rate was higher in historical controls (36%) than intervention (24%) (p-value = 0.35), and a similar trend was observed in myeloid malignancy patients. EBV reactivation rate was similar in historical controls (33%) and in the Vitamin C recipients (35%) (p-value = 0.88). There were no grade 3 – 5 adverse events attributable to vitamin C therapy in this trial, particularly no nephrotoxicity or renal calculi were observed.

### Cytokine levels in Vitamin C recipients

**Figure 5:**
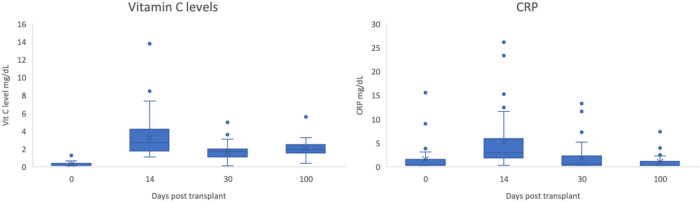
(A) Vitamin C levels in the entire cohort of patients. (B) CRP levels at the same time points.

Vitamin C level (normal 0.4–2.0 mg/dL) was universally low prior to intervention and was restored to normal by day 14 (Figure 5), while CRP (normal 0–0.5 mg/dL) rose in the early aftermath of transplant following conditioning and engraftment to come down later. The peaks for both the parameters coincided, suggesting potential benefit of vitamin C may have come from its physiologic effect at the time of maximum inflammatory cytokine surge. The first 14 patients treated with vitamin C had pro-inflammatory cytokines IL-1b, IL-2, IL-6, IL-12, TNF-α, IFN-γ, and soluble thrombomodulin measured, which remained unchanged between day 0 and day 14 & day 30 after HSCT (P=NS for all direct comparisons). Heat maps demonstrating change of cytokine levels post-transplant showed that most of the patients showed either a reduction or stabilization of their inflammatory cytokine levels following SCT, with few patients demonstrating an increase at all the time points tested following HSCT (**Supplementary Figure 3**). However, in most instances the cytokine levels were back down in the lower ranges by day 30. Elevated cytokine levels on day 14 appear to correspond to higher magnitude of donor derived T cell recovery by day 30 following transplant (**Supplementary Figure 4**).

## DISCUSSION

In this paper the results of a prospective trial evaluating parenteral vitamin C in allogeneic HSCT are presented, demonstrating its safety. There is a non-relapse mortality and survival benefit observed, especially in recipients with myeloid malignancies when compared with propensity-matched historical controls, adjusting for diagnosis, disease risk, and conditioning intensity.

Non-relapse mortality is the Achilles heel of allografting, compromising the benefit that comes from the immune graft vs. malignancy effect. The foundation for NRM is laid in the initial mucosal and endothelial injury seen following high-dose therapy, which sets the stage for alloantigen presentation to donor cells, setting up the cascade of alloimmune response culminating in GVHD.^12, 13^ Pro-inflammatory cytokines released following tissue injury from high-dose therapy is a major contributor to the pathophysiology in these instances.^14^ This is similar to sepsis, where parenteral vitamin C has been shown to ameliorate endothelial injury, especially in lung injury and acute respiratory distress syndrome. ^8^ Further due to the oxidative stress of pretransplant conditioning with radiation and alkylating agents,^15 16^ followed by poor oral intake, the vitamin C stores are rapidly depleted,^17^ leading to a severely deficient state. ^11 18 19^ Given this logic the clinical trial reported here was designed to rapidly restore vitamin C following transplantation and utilize its endothelial stabilizing effect to reduce the impact of mucosal and endothelial injury post-transplant.^20 21^ Ameliorating the endothelial injury in turn modulates the inflammatory milieu, potentially mitigating alloantigen presentation in the early days following transplant, eventually reducing GVHD likelihood evolving from recovering donor derived T cells. This concept is supported here by the observed trend toward reduced acute GVHD (grade II-IV) and chronic GVHD in Vitamin C recipients. This reduction in chronic GVHD incidence was observed in vitamin C recipients compared to historical controls despite an equivalent number of HLA MUD and PBSC recipients, and a different schedule of ATG administration designed to enhance donor T cell recovery.^22^ Despite the reduction in chronic GVHD, no worsening of relapse risk was observed, even though a higher proportion of Vitamin C recipients underwent conditioning with fludarabine and melphalan 140mg/m^2^, a reduced intensity regimen. This points to a potential modulation of disease risk impact in addition to attenuating GVHD in these high-risk leukemia patients.

Vitamin C is a cofactor for TET-2 enzyme, and may have a role in reducing relapse risk in patients with mutation impacting this mechanism of hypomethylation.^23^ Vitamin C by helping restore the oxidative function of TET-2 would reduce the risk of relapse in myeloid malignancies. ^24 25 26 27^ While the relapse rate is not significantly different in this cohort, those relapsing had high-risk disease, and included patients who were transplanted with active disease and primary induction failure. A further confounding factor was that most patients underwent GVHD prophylaxis with ATG, potentially limiting the GVL effects that may be observed. One may surmise that vitamin C mediated relapse protection effect may be most evident in patients who do not get in vivo T cell depletion. This will also be the population where any GVHD benefit from diminished alloantigen presentation may be best observed.

A reduction in inflammation may increase the risk of infections. Infections were seen in the vitamin C recipients, but not at a higher rate than the matched historical controls, nevertheless given a reduction in the inflammatory response, this bears close observation in future trials, as was suggested by a randomized trial of vitamin C administration in patients with early sepsis.^28^ Such a trial will, of necessity, must be randomized, and optimally conducted in patients who do not receive T cell depletion (in vivo or ex vivo). Parenteral vitamin C has been reported to lead to an improvement in biomarker profile in sepsis, consistent with that, this study also demonstrated an amelioration of adverse cytokine profiles following high-dose therapy and HSCT.

If vitamin C repletion following high-dose therapy and HSCT is proven to be of benefit in patients undergoing T cell replete allografts and reduce relapse risk, it will be a significant advance on a global scale, particularly in economically challenged regions, given its low cost and wide availability. Therefore, our study demonstrating the absence of increased toxicity and impact on GVHD, relapse and non-relapse mortality represents a crucial first step in that direction.

## Figures and Tables

**Table 1:** Summaries for Matching Variables and Other Patient Measurements (before and after Matching)

		Before Matching		After Matching	
		Historical Control	Intervention	Historical Control	Intervention
**Sample Size**		70	55	55	55

**Disease Type**	ALL	19 (27%)	14 (25%)	16 (29%)	14 (25%)
	AML	32 (46%)	25 (45%)	24 (44%)	25 (45%)
	CML + MDS	19 (27%)	16 (29%)	15 (27%)	16 (29%)

**Conditioning Regimen**	BU-CY	36 (51%)	11 (20%)	21 (38%)	11 (20%)
	FLU-MEL	20 (29%)	30 (55%)	20 (36%)	30 (55%)
	TBI-CY	14 (20%)	14 (25%)	14 (25%)	14 (25%)

**Disease Risk**	High	43 (61%)	41 (75%)	41 (75%)	41 (75%)
	Intermediate + Low	27 (39%)	14 (25%)	14 (25%)	14 (25%)

		Matched Historical Controls (N=55)		Matched Intervention (N=55)	
		Mean	SD	Mean	SD
	Age	50.6	11.7	53.3	11.2

		Frequency	%	Frequency	%
**Donor Type**	MRD	14	25%	11	20%
	MUD	41	75%	44	80%

**Stem Cell Source**	BM	6	11%	2	4%
	PBSC	49	89%	53	96%

**Fatality**	Yes	21	38%	10	18%

**Relapse**	Yes	13	24%	12	22%

**Non-Relapse Mortality**	Yes	14	25%	6	11%

**Non-GVHD-Relapse Mortality**	Yes	4	7%	3	6%

**Acute GVHD (Stage II-IV)**	Yes	18	33%	18	33%

**Acute GVHD (Stage III-IV)**	Yes	13	24%	9	17%

**Chronic GVHD**	Yes	13	24%	6	11%

**CMV Reactivation**	Yes	20	36%	14	25%

**EBV Reactivation**	Yes	18	33%	19	35%


**Table 2:** Summaries for Matching Variables and Other Patient Measurements (before and after Matching)

		Before Matching		After Matching	
		Historical Control	Intervention	Historical Control	Intervention
**Sample Size**		51	41	41	41

**Disease Type**	AML	32 (63%)	25 (61%)	24 (59%)	25 (61%)
	CML + MDS	19 (37%)	16 (39%)	17 (41%)	16 (39%)

**Conditioning Regimen**	BU-CY	33 (65%)	11 (27%)	23 (56%)	11 (27%)
	FLU-MEL	18 (35%)	28 (68%)	18 (44%)	28 (68%)
	TBI-CY	0 (0%)	2 (5%)	0 (0%)	2 (5%)

**Disease Risk**	High	30 (59%)	31 (76%)	30 (73%)	31 (76%)
	Intermediate + Low	21 (41%)	10 (24%)	11 (20%)	14 (24%)

		Matched Historical Controls (N=41)		Matched Intervention (N=41)	
		Mean	SD	Mean	SD
	Age	52.8	11.6	55.6	10.0

		Frequency	%	Frequency	%
**Donor Type**	MRD	12	29%	9	22%
	MUD	29	71%	32	78%

**Stem Cell Source**	BM	4	10%	2	5%
	PBSC	37	90%	39	95%

**Fatality**	Yes	19	46%	7	17%

**Relapse**	Yes	10	24%	8	20%

**Non-Relapse Mortality**	Yes	15	37%	4	10%

**Non-GVHD-Relapse Mortality**	Yes	4	10%	1	3%

**Acute GVHD (Stage II-IV)**	Yes	17	41%	15	38%

**Acute GVHD (Stage III-IV)**	Yes	14	34%	9	23%

**Chronic GVHD**	Yes	10	24%	2	5%

**CMV Reactivation**	Yes	19	46%	12	29%

**EBV Reactivation**	Yes	14	34%	13	32%

